# Hidden female physiological resistance to male accessory gland substances in a simultaneous hermaphrodite

**DOI:** 10.1242/jeb.149963

**Published:** 2017-03-15

**Authors:** Monica Lodi, Joris M. Koene

**Affiliations:** 1 Section of Animal Ecology, Department of Ecological Science, Faculty of Earth and Life Sciences, VU University Amsterdam, De Boelelaan 1085, Amsterdam 1081 HV, The Netherlands; 2 Naturalis Biodiversity Center, Vondellaan 55, Leiden 2332 AA, The Netherlands

**Keywords:** Allohormone, Antagonistic coevolution, Diverticulum, Love-dart, Sexual conflict, Sexual selection

## Abstract

To increase fertilization chances compared with rivals, males are favoured to transfer accessory gland proteins to females during mating. These substances, by influencing female physiology, cause alteration of her sperm usage and remating rate. Simultaneously hermaphroditic land snails with love-darts are a case in point. During courtship, a love-dart is pierced through the partner's body wall, thereby introducing accessory mucous gland products. This mucus physiologically increases paternity by inhibiting the digestion of donated sperm. The sperm, which are packaged in a spermatophore, are exchanged and received in an organ called the diverticulum. Because of its length, this organ was previously proposed to be a female anatomical adaptation that may limit the dart interference with the recipient's sperm usage. For reproductive success of the donor, an anatomically long spermatophore, relative to the partner's diverticulum, is beneficial as sperm can avoid digestion by exiting through the spermatophore's tail safely. However, the snail *Eobania vermiculata* possesses a diverticulum that is three times longer than the spermatophore it receives. Here, we report that the love-dart mucus of this species contains a contraction-inducing substance that shortens the diverticulum, an effect that is only properly revealed when the mucus is applied to another helicid species, *Cornu aspersum*. This finding suggests that *E. vermiculata* may have evolved a physiological resistance to the manipulative substance received via the love-dart by becoming insensitive to it. This provides useful insight into the evolution of female resistance to male manipulations, indicating that it can remain hidden if tested on a single species.

## Abstract (precis)

**Summary:** The effect of accessory gland proteins transferred on the love-dart of land snails can remain hidden within a species as a result of physiological female resistance to male manipulation.

## INTRODUCTION

Males are generally in competition for reproductive access to females prior to copulation (Andersson, 1994). However, when females mate promiscuously, ejaculates of rival males can compete for egg fertilization inside the female reproductive system, referred to as sperm competition (Parker, 1970). This form of sexual selection and the potential selection on females to selectively use sperm for fertilization (the latter being referred to as cryptic female choice) are two evolutionary drivers that are difficult to separate as they both predict differential use of ejaculates in fertilization and favour post-copulatory adaptations that ultimately increase reproductive success (Eberhard, 1996; Birkhead and Pizzari, 2002; Snook, 2005; Orr and Brennan, 2015). Examples of these adaptations, in males, include increased sperm mobility to reach the sperm-storage organ faster (e.g. domestic fowl; Birkhead et al., 1999), larger testes size to transfer more sperm under strong competition (e.g. frog *Crinia georgiana*; Dziminski et al., 2010) and transfer of accessory gland substances that influence female physiology (reviewed by Gillott, 2003). Common effects include reduced female remating rate to enhance storage of donated sperm (e.g. fruit fly *Drosophila melanogaster*; Chapman and Davies, 2004) and altered sperm usage by inducing oviposition following mating (e.g. cotton bollworm *Helicoverpa armigera*; Jin and Gong, 2001). In response, females may physiologically resist such male manipulations by modifying the targeted receptors or by increasing the threshold at which male products are effective (Holland and Rice, 1998; Rowe et al., 2003). This could occur in response to sexual conflict that leads to an arms-race between male adaptations and female counter-adaptations (Arnqvist and Rowe, 2002), by which females reduce the direct costs imposed by the manipulation and can select for males that are able to overcome such resistance (Cordero and Eberhard, 2003; Eberhard, 1996, 2009). However, in this context it is useful to note that female choice, in general, can vary plastically over a reproductive season or life time (e.g. Lynch et al., 2005), thus adding a layer of complexity to the interpretation.

Just like in many separate-sexed organisms, sperm of different donors can also co-occur in the female reproductive system of simultaneous hermaphrodites (i.e. organisms with functioning male and female reproductive organs at the same time) as these organisms also mate promiscuously (Baur, 1998; Michiels, 1998; Koene, 2017). The male function of simultaneous hermaphrodites also transfers accessory gland substances that physiologically affect sperm usage by the mating partner (Zizzari et al., 2014). An interesting case is presented by the love-dart of land snails. This accessory reproductive device is a calcareous stylet with a species-specific shape (reviewed by Lodi and Koene, 2016b). The love-dart pierces the partner's body wall during courtship, in a behaviour called dart shooting (Tompa, 1984), while holding accessory mucous gland products on its surface. In the model species the brown garden snail *Cornu aspersum*, the mucus that enters the partner's haemolymph has been shown to cause two temporary changes in the female reproductive system (Koene and Chase, 1998; for a visualization of the effects, see supplementary movie 1 in Lodi and Koene, 2016a). The first change can be measured as waves of muscular contractions that close the entrance to the sperm-digesting organ, the bursa copulatrix, and have been shown to delay sperm digestion. As a result, more sperm are stored and the successful dart user can double its paternity (Chase and Blanchard, 2006). The second change involves the diverticulum, the spermatophore-receiving organ (i.e. the organ that receives the package containing sperm). Under the influence of dart mucus, contractions of this blind-ended duct are initiated, possibly making spermatophore uptake easier (Koene and Chase, 1998).

**Fig. 1.**
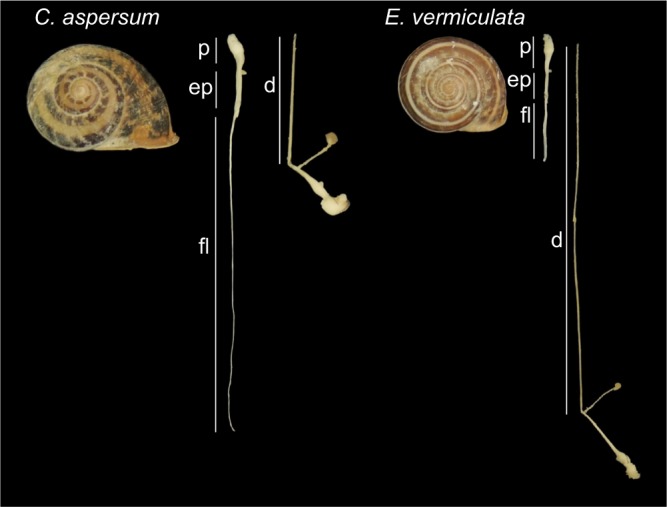
**Spermatophore-producing and -receiving organs of *Cornu aspersum* and *Eobania vermiculata*.** Shell length is 3.5 cm for *C. aspersum* and 2.6 cm for *E. vermiculata*. d, diverticulum; ep, epiphallus; fl, flagellum; p, penis.

In this scenario, the female function of dart-bearing snails may resist the manipulation of the dart. So far, only morphological co-evolutionary patterns have been shown by an inter-species comparison (Koene and Schulenburg, 2005). That study found that in species where the shape of the dart increased the surface available to transfer mucus (e.g. by blades and perpendicular blades), the diverticulum appeared and became longer. Thus, the diverticulum has been proposed to be a female anatomical adaptation to counter dart manipulation. This is also based on the finding that sperm need to leave safely through the spermatophore's tail when it protrudes from the diverticulum entrance into the vaginal duct in order to reach the site of storage (Lind, 1973), so the length of the diverticulum may limit the ability of the dart to interfere with the process of sperm digestion. This idea is strengthened by the positive correlation found between the length of the spermatophore's tail and the diverticulum (Koene and Schulenburg, 2005). However, resistance to the dart probably also extends to the physiological and biochemical level. A recent cross-reactivity test between species showed that the dart mucus of *Eobania vermiculata* caused a temporary contraction of the diverticulum of *Cornu aspersum* that reduced its length by approximately 20% (for a visualization of this effect, see supplementary movie 2 in Lodi and Koene, 2016a). Both species belong to the Helicidae family and are relatively closely related, but they differ markedly in the length of their spermatophore-producing organs and diverticula. Unlike that of *C. aspersum*, the diverticulum of *E. vermiculata* is extremely long (see Fig. 1; Table S1), which is an exception to the above-mentioned general correlation (Koene and Schulenburg, 2005). Hence, to avoid donated sperm being digested by the mating partner, the male function of *E. vermiculata* would require either the spermatophore to become elongated or the organ in which it is received to be shortened. In our previous study, we showed that in this species the latter option is achieved via an effect that shortens the diverticulum (Lodi and Koene, 2016a).

However, in our previous study we focused on responses of the *C. aspersum* reproductive organs in order to determine how conserved the effects of the dart mucus are, so we did not test whether such female physiological responses also occurred in *E. vermiculata*. Thus, to investigate this prediction, we here studied the extent of the shortening effect on the diverticulum of *C. aspersum* by performing a dose–time response test. Subsequently, we assessed whether this effect is specifically caused by mucus carried on the love-dart and, importantly, whether the shortening response also occurs in *E. vermiculata*.

## MATERIALS AND METHODS

Adult snails of the species *Cornu aspersum* (O. F. Müller 1774) and *Eobania vermiculata* (O. F. Müller 1774) were obtained from a snail farm (Euro Helix, Cherasco, Italy). The rearing conditions were 20°C, a reversed photoperiod (16 h light:8 h dark) and 60% humidity. Twice a week, the snails were cleaned, fed lettuce *ad libitum* and snail feed as a source of calcium (‘Chase’ mix; see Lodi and Koene, 2016a). Before experiments began, snails were kept individually for at least 2 weeks on moist paper in plastic boxes (11.5×11.5×5 cm) to ensure that mucus would be available in the glands for the tests. The set-up used was a portion of the reproductive system of either species, hereafter called ‘preparation’ (for a visualization, see Lodi and Koene, 2016a), kept in a small dish with 2 ml saline solution at pH 7.8 (control saline; Goldstein et al., 2000), which corresponds to the volume of haemolymph of both species (*C. aspersum*, Martin et al., 1958; *E. vermiculata*, Beltagi et al., 2011). The preparation was obtained by anaesthetizing a snail with 50 mmol l^−1^ MgCl_2_ and dissecting out the genital atrium, copulatory canal, diverticulum, and bursa copulatrix and its tract. Immediately after dissection, the preparation was allowed to equilibrate in the saline bath for 30 min and between trials it was rinsed three times with saline solution and left for 5 min in fresh saline. Each trial consisted of the addition of an extract or saline solution (control) to the saline bath. Extracts were obtained each day by dissecting the tissue of interest out of one snail and crushing it with a plastic pestle in 0.5 ml saline. For each test, one preparation was used per day for testing in all different trials and each extract was used once to avoid pseudoreplication. To assess the length of the diverticulum, pictures of the preparation were taken with a webcam (Logitech^®^ HD Pro Webcam c920). Diverticulum length was measured in ImageJ from its tip to the base where the bursa copulatrix tract branches. For each flank of the organ, three measurements were made and the average of these considered.

### Dose–time test

To test the extent of the shortening effect, we performed a dose–time test on *C. aspersum*. Preparations of this species (*N*=10) were each tested with five test substances in random order. These trials included four doses of accessory mucous gland extracts of *E. vermiculata* and one dose of saline solution. The mucus doses were equivalent to 1.1, 2.2, 3.3 and 4.4 mg of the extract. Note that 2.2 mg is roughly the maximum amount of mucus that the love-dart of *C. aspersum* can carry (calculated as the difference between the wet mass of shot and non-shot darts; Koene and Chase, 1998); 48 µl of saline was added, which equals the average volume of 2.2 mg mucus. A picture of the preparation was taken before addition of a substance (time 0 min) and successively every 5 min for 30 min. In total, the amount of time each preparation was used is comparable to that employed in the experiments of Koene and Chase (1998): approximately 3.5 h.

### Occurrence and specificity of shortening effect

To test whether the shortening effect is specifically caused by *E. vermiculata*'s dart mucus, we applied this and several other substances to preparations of *C. aspersum* (*N*=18). The tested extracts were accessory mucous gland extracts of *C. aspersum* and *E. vermiculata* as well as body wall extracts from both species. Both materials are most likely to be introduced into a mating partner of the same species during dart shooting. When the dart perforates the partner, some of the mucus present on the body surface and mucus contained in the mucous cells embedded in the epithelium (Luchtel et al., 2001) probably enter the snail, although in insignificant quantities compared with the mucus covering the dart. The body wall for the extract was taken from the area next to the genital pore of both species, where the dart normally hits the partner (Lodi and Koene, 2016b). As a control, 48 µl of saline was used. The amount of mucus and body wall extract added was equivalent to 2.2 mg, as it is the maximum dosage that a partner would receive (Koene and Chase, 1998). Two pictures of the preparation were taken: before and 5 min after addition of each substance (this was a sufficient amount of time to see a distinct effect from saline according to the results of the dose–time test).

To test whether the shortening effect also occurred when mucus of *E. vermiculata* was applied to its own reproductive system and whether it was specific to dart mucus, *E. vermiculata* preparations (*N*=18) were tested with the five above-mentioned substances by following the same protocol.

### Statistical analyses

Data were log-transformed only for tests on the occurrence and specificity of the shortening effect on *C. aspersum*, as not all groups were normally distributed. For all tests, a mixed ANOVA was performed to estimate the effect of time and treatment on diverticulum length. This test compares means between two or more independent variables and one of them can be a repeated measure (Field, 2005). In our case, time and treatment were the two independent variables and time was the repeated measure, as the diverticulum length of each preparation was measured at two or more time points. When the sphericity assumption was violated (only for the dose–time test), we performed the Greenhouse–Geisser correction. If the interaction time×treatment was significant, the simple effect with Fisher's LSD adjustment was tested. This test assesses whether a variable has a significant effect at each level of another variable by making pairwise comparisons. For the dose–time test, we also calculated percentages of length gain or reduction per time point compared with the value at 0 min to see whether the shortening response increased over time. In addition, to test for dependency in our data (as one preparation was used per day to test all treatments), for all tests we performed one-way ANOVA on diverticulum length at time 0. If the original length was regained after each trial, the measurements returned to the same baseline.

## RESULTS

### Dose–time test

**Fig. 2.**
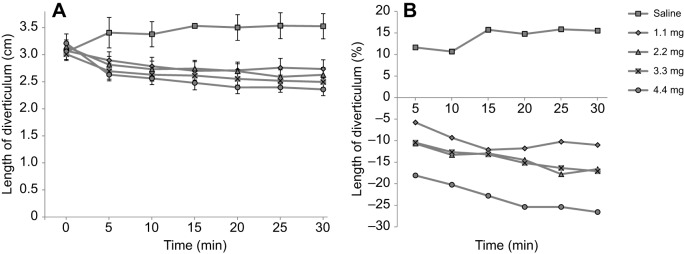
**Dose–time response of *C. aspersum*'s diverticulum.** (A) Mean (±s.e.) length of the diverticulum, which became shorter when love-dart mucus of *E. vermiculata* was applied at four different doses, and gained length when saline solution was applied as a control. (B) Diverticulum length gain or reduction as a percentage of the length at the zero time point (0 min).

After applying four doses of *E. vermiculata*'s dart mucus and one dose of saline to the *C. aspersum* preparation, time and the interaction time×treatment were significant (*F*_2.5_=21.877, *P*<0.001; *F*_10_=9.164, *P*<0.001, respectively). This implies that the different tested substances did not all cause a reaction in the same way over time. At 5 min, all mucous doses significantly differed in comparison to saline (all *P*<0.05), except for the lowest dose, which showed a trend in the same direction as the other doses (*P*=0.052). This means that the mucous extracts induced length reduction compared with the control depending on the dose (Fig. 2A). At 30 min, all the doses differed from saline (all *P*<0.01), indicating that the shortening effect remained effective over time. Percentages of diverticulum length gain or reduction per time point compared with the value at 0 min are indicated in Fig. 2B, which shows that with saline addition the diverticulum relaxed over time, increasing in length, while the mucous doses induced the diverticulum to become shorter and this response increased over time. For all measurements, the diverticulum regained its original length after each trial (*F*_4,45_=0.295, *P*=0.880).

### Occurrence and specificity of shortening effect

**Fig. 3.**
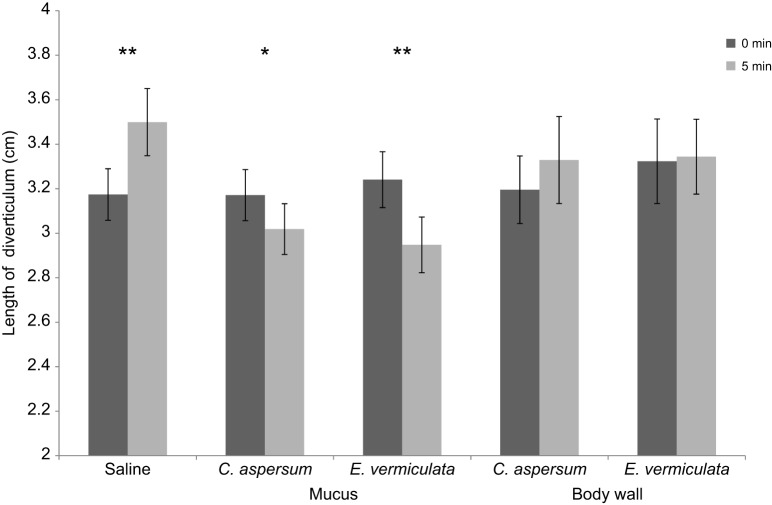
**Length of *C. aspersum*'s diverticulum in response to different substances.** Mean (±s.e.) length is shown, measured before (0 min) and after (5 min) the addition of the five tested substances. *P<0.05, **P<0.001.

When five different substances were applied to the *C. aspersum* preparation, there was a significant time×treatment interaction (*F*_4_=9.622, *P*<0.001). Comparison of the response among treatments over time shows that the saline solution induced the diverticulum to relax (*P*<0.001); neither body wall extract induced the diverticulum to become shorter (*C. aspersum*: *P*=0.177; *E. vermiculata*: *P*=0.703) but the mucous extracts did (*C. aspersum*: *P*=0.036; *E. vermiculata*: *P*<0.001) (Fig. 3). Between the two species, *E. vermiculata* mucus showed the strongest effect by decreasing the diverticulum by 9% of its original length compared with approximately half the length reduction (4.8%) by *C. aspersum* mucus.

In contrast, *E. vermiculata*'s diverticulum showed no shortening effect in response to any of the five substances that were applied (time: *F*_1_=0.119, *P*=0.732; time×treatment: *F*_4_=0.897, *P*=0.470) (Fig. S1). For both species, the diverticulum regained its original length after each trial (*C. aspersum*: *F*_4,85_=0.130, *P*=0.971; *E. vermiculata*: *F*_4,85_=0.350, *P*=0.844).

## DISCUSSION

Dart-bearing land snails affect the way in which their donated sperm are used by the partner via accessory mucous gland products transferred during courtship (Koene and Chase, 1998; Kimura et al., 2013, 2014; Lodi and Koene, 2016a). Our study reveals, for the first time, that resistance to manipulation induced by these substances can occur at the physiological level, rather than only anatomically. This is only detectable when the love-dart mucus of *E. vermiculata* is applied to another helicid, *C. aspersum*. In this case, the length reduction of the diverticulum is visible and induced in a dose–time-dependent fashion, becoming more effective over time. This shortening effect is most strongly caused by *E. vermiculata*'s dart mucus.

Morphologically, *E. vermiculata* has two ways in which it could enable the spermatophore's tail to protrude into the vaginal duct of its mating partner, thus avoiding digestion of donated sperm (Lind, 1973): either the spermatophore length would need to be increased or the organ in which it is received would need to become shorter. For *C. aspersum*, the first option seems to apply as its spermatophore is more than twice the diverticulum length (see Fig. 1; Table S1). However, the opposite situation occurs in *E. vermiculata*: the spermatophore represents only a third of the length of the diverticulum (see Fig. 1; Table S1). Given the difficulty that this long organ might impose on sperm survival is thus not overcome anatomically, the evolution of a substance that shortens the diverticulum physiologically would be beneficial as this would reduce the distance to the oviduct entrance. The diverticulum has the potential to be modified in such a way because it is a very flexible organ. For example, its wall has been reported to change in thickness and diameter following spermatophore receipt (Beese et al., 2006) and in our tests it regained its original length after the dart mucus was washed away.

Interestingly, our results indicate that *E. vermiculata* is insensitive to the shortening effect caused by its own mucus. This may be due, for example, to a reduction in the receptor sensitivity on this species' diverticulum to the shortening-inducing substance. This could be interpreted as evidence of antagonistic co-evolution to the manipulative effect caused by the love-dart mucus. If this is the case, the results would indicate that the female function gained an advantage in the conflict for this particular effect (Arnqvist and Rowe, 2002). However, note that this scenario applies provided that the love-dart imposes costs on the dart receiver, thereby causing sexual conflict (Arnqvist and Rowe, 2002, 2005). Co-evolution between male and female traits can generally also be explained by other sexual selection processes (e.g. female choice, Fisherian run-away and good-genes/sexy sons) that are not necessarily mutually exclusive (Andrés and Arnqvist, 2001; Arnqvist and Rowe, 2005; Rowe and Day, 2006; Rowe et al., 2003). To disentangle these, the costs and benefits, to the female, of being manipulated should be quantified (Eberhard, 2009).

In contrast, *C. aspersum* never had the opportunity to receive mucus from *E. vermiculata*, and hence it did not evolve resistance to that particular substance. Thus, we were able to detect this expected hidden female resistance in *E. vermiculata* only by testing the two species for cross-reactivity. One might also have expected *E. vermiculata* to react to *C. aspersum*'s mucus, as the former also did not have the opportunity to co-evolve with the latter's mucus substance. However, as the shortening effect on *C. aspersum*'s diverticulum is induced by the mucus of both species, there is the possibility that these substances are similar (see also Lodi and Koene, 2016a). If this is the case, *E. vermiculata* becoming insensitive to its own substance may, as a result, also prevent a reaction to *C. aspersum*'s dart mucus. Further research on the identification of such substances, and their receptors, would clarify this point.

Although *E. vermiculata* did not display the shortening effect, this mucus effect still persists even though it seems to bring no benefit to the dart user, based on the results obtained so far. This situation could be explained if the female function recently evolved this resistance and the male has not yet counter-adapted. Alternative explanations could be that the manipulative substance also causes other effects in the dart recipient that were not measured here, or that there are no significant costs for producing this particular substance. Despite not showing this shortening effect, increased male reproductive success in *E. vermiculata* can still be achieved by other post-copulatory mechanisms, such as the closing off of the entrance to the bursa copulatrix (Koene and Chase, 1998; Lodi and Koene, 2016a). Other common strategies in the animal kingdom in this context are, for example, the evolution of fast-swimming sperm (e.g. cichlids; Fitzpatrick et al., 2009) and long sperm (e.g. stalk-eyed flies; Presgraves et al., 1999), and the addition of new manipulative substances in the seminal fluid (Eberhard, 1996).

In general, our results are comparable to cross-population studies on signal–receptor co-evolution between sexes in flies, which also suggests evolution of female resistance. For example, during mating, males of the housefly *Musca domestica* transfer accessory gland substances (seminal fluid proteins) that induce oviposition in females (Riemann and Thorson, 1969). However, when females mate with males of different strains (compared with their own), they show an increased oviposition effect (Andrés and Arnqvist, 2001). Another example is the seminal fluid of male *Drosophila melanogaster*, which is able to remove sperm of males that previously mated with the female (Harshman and Prout, 1994). This effect is weaker in females of the same strain (Clark et al., 1999). Strong resistance to the same male strain may occur as a result of antagonistic co-evolution between female receptors and male seminal products (Andrés and Arnqvist, 2001; Rice, 1996). However, this is not always the case when crossing strains or populations (Chapman et al., 2003; Rowe et al., 2003).

In summary, despite the abundance of simultaneous hermaphrodites (Anthes, 2010), little is known about the consequences of the conflict over usage of donated sperm between mating partners that have both sexual functions present in the same body (reviewed by Schärer et al., 2014). Until recently, studies on separate-sexed species have dominated this field of research (e.g. Friesen et al., 2016) and, as a result, explanations based on separate sexes have dictated theory so far. Our current findings are in agreement with those observed in sexual antagonistic co-evolution in separate-sexed species. However, they do highlight that selection outcomes can be complicated and conceptually more difficult to comprehend in organisms that are male and female at the same time (Koene, 2017), as simultaneous hermaphrodites express both the genes for producing manipulative compounds and those for resisting their effects. Finally, our study is the first to provide evidence for simultaneous hermaphrodites that is in line with the idea that female resistance to a male signal may take place at the physiological level and, more importantly, that it can remain hidden when tested on a single species.
